# The Multiple Roles of Genetics on Freshwater Macrophyte Functional Traits in the Interplay With the Environment: A Review

**DOI:** 10.1002/ece3.74326

**Published:** 2026-09-06

**Authors:** Beatrice Fois, Alice Dalla Vecchia, Giovanni Campiello, Rossano Bolpagni, Carla Lambertini

**Affiliations:** ^1^ Department of Biosciences University of Milan Milan Italy; ^2^ Department of Chemistry, Life Sciences and Environmental Sustainability University of Parma Parma Italy; ^3^ Department of Biology McGill University Montreal Canada

**Keywords:** adaptation, environmental heterogeneity, epigenetics, genetic drift, genetic variation, genome size, phenotypic plasticity, selection

## Abstract

The study of functional trait variation is increasingly used to understand macrophyte adaptation, as traits reflect organismal performance under different ecosystem conditions. Phenotypic expression results from the interplay of genetic and environmental factors: genetics provides the molecular basis for heritable traits and constrains potential phenotypes, while the environment acts as a selective and modulatory force. However, the genetic insight into traits has rarely been addressed in freshwater macrophyte studies. This review examines the different ways in which the DNA of macrophytes interplays with the environment and contributes to the variation in their functional traits, outlining main approaches, gaps, and future challenges. Only 21 studies explicitly combined genetics with functional traits and environment in the last fifteen years. The most common approach was the use of common garden experiments to explore acclimation and adaptation in a few model species. Current studies mainly focus on morphological and growth traits that best describe macrophytes' economic strategies, with limited attention to other trait categories, while the genetic and DNA traits studied are more variable. Across studies, environmental factors generally explained a larger proportion of functional trait variation, highlighting the dominant role of phenotypic plasticity for macrophyte acclimatation, whereas genetic contribution increased under experimentally manipulated conditions. Genome size and epigenetic variation influenced phenotypic plasticity; however, the effect was different and inconsistent on traits and depended on phylogenetic relationships and geographical environment variation. In field studies of natural populations, life history traits and hydrology had a strong effect on the geographic distribution of genetic diversity and the response to selection, as well as on our ability to distinguish selection from genetic drift. Future research should enhance molecular analyses, adopt multifactorial and long‐term experimental designs, develop conceptual frameworks to address the relationships between genomics, environment and functional traits and integrate emerging tools to capture macrophyte adaptation better.

## Graphical abstract

This review examines how genetic and environmental factors jointly shape functional trait variation in aquatic plants. By synthesizing the literature from the last fifteen years, we analyze the roles of genetic diversity, epigenetic variation, genome size, and gene expression in relation to phenotypic plasticity, natural selection, and genetic drift. We highlight major knowledge gaps and outline future research directions to better understand the evolutionary significance of functional trait variation in macrophytes.

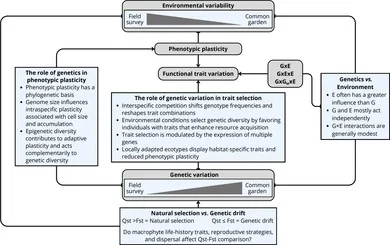

## 1 Introduction

Macrophytes are an important component of freshwater ecosystems, playing a key role in aquatic food webs and bio‐geochemical cycles (Benelli and Bartoli 2021; Lind et al. 2022; Scorsim et al. 2024). They influence the hydrology and sediment dynamics of aquatic habitats by altering current velocity, attenuating light penetration in the water column, providing habitat and refuge to many living organisms, from microbiota to vertebrates, and offering multiple benefits to humans (Bornette and Puijalon 2011; Thomaz 2023). Considering the significant role macrophytes play in aquatic ecosystems, it is important to understand the factors that determine their performance and distribution patterns, reflecting their ability to cope with a rapidly changing environment (Lind et al. 2022).

A popular approach to examine macrophytes' response to the environment is through functional traits (Dalla Vecchia et al. 2020), which are defined as any morphological, physiological and phenological feature of plants (Diaz et al. 1998; Pierce et al. 2013). Plant functional traits are thought to profoundly affect plant fitness, directly or indirectly, since they reflect organism performance and life history strategy in response to environmental variation (Diaz et al. 2004; Islam et al. 2024; Ren et al. 2020). Therefore, they represent a useful tool to explore and predict many ecological aspects, including community assembly, species responses to environmental gradients and impacts on ecosystem processes (Chelli et al. 2019; Liu et al. 2024) and intraspecific trait variation, that can be considerably high for freshwater plants, as aquatic environments are very dynamic (Dalla Vecchia and Bolpagni 2022). Freshwater plant traits can change rapidly among populations and at relatively narrow spatial scales in response to many abiotic and biotic factors, such as hydrological conditions, pollutants and competition (Lacoul and Freedman 2006; Cao et al. 2012). For instance, some plants develop differently shaped leaves (heterophylly) when they are below or above water surface (Wells and Pigliucci 2000; Vretare et al. 2001; Cao et al. 2012; Molnár et al. 2015). The integration of functional traits into biodiversity monitoring has improved our ability to understand and quantify differences among environments and plant performance. This information provides valuable insights for conservation and restoration actions, while also advancing our understanding of the eco‐evolutionary processes underlying phenotypic variation (Wright et al. 2016; Born and Michalski 2017).

The plant phenotype is the result of environment and genetics and is variable among individuals because of different DNA sequences (Albert et al. 2011; Wright et al. 2016). Genetic variation refers to the diversity in DNA sequences among individuals within a species (Albert et al. 2011). It arises from processes that generate diversity like meiosis, sexual recombination, DNA mutation, and the dispersal and establishment of genes through pollen and seeds within and among populations (gene flow). Natural selection and/or stochastic processes remove or fix this variation across populations. Natural selection acts on specific genes responsible for environment‐adaptive traits, whereas in small‐size and isolated populations stochastic phenomena can change allele frequencies independently of the environment (genetic drift). On the other hand, gene flow can homogenize the variation within and between randomly interbreeding populations (Ulukapi and Nasircilar 2018). Genetic variation is fundamental for population fitness because among the different individuals of a population some can resist environmental stressors and adapt to changing conditions. High levels of genetic variation are not very common for freshwater plants that differ considerably from the terrestrial species in this respect, as vegetative propagation through shoot fragmentation is often the most efficient and common form of dispersal for submersed and floating plants (Barrett‐Lennard 1993; Pollux et al. 2007). Sexual reproduction can be problematic because of the spatial and temporal heterogeneity of suitable aquatic habitats, as even small‐scale fluctuations in water level, temperature, stream velocity and nutrient circulation may disrupt flower and seed production, fruit maturation, germination, or seedling establishment (Barrett‐Lennard 1993; Engloner et al. 2023). In rivers, in addition, unidirectional hydrochory may increase genetic diversity from upstream to downstream populations and, for this reason, some authors suggested that downstream populations can adapt better than upstream populations to natural selection (Liu, Wang, et al. 2021; Liu, Yin, et al. 2021). Dams, hydrological conditions and different buoyancy of seeds can also affect seed transport and deposition in water bodies and these factors further affect the distribution of genetic diversity and ultimately the evolutionary processes that can take place (Barrett‐Lennard 1993; Sand‐Jensen et al. 1999; Pollux et al. 2007). Plant fragments, on the other hand, can be dispersed over large distances and can establish new big clonal populations. Examples of the efficiency of clonal dispersal come from the invasive species *Elodea canadensis* , *Egeria densa* , and *Lagarosiphon major* that in the introduced range in New Zealand have one single genotype (Lambertini et al. 2010; Huotari and Korpelainen 2013). Interestingly, these clonal populations are neither less fit than those of species with large genetic diversity, nor are less variable in functional traits (Riis et al. 2010), suggesting either an exclusive contribution of the environment to their phenotypic traits or that molecular factors other than genetic variation are important for their phenotypic variation.

Phenotypic plasticity, which is defined as the ability of a genotype to produce distinct phenotypes under different environmental conditions (Schlichting 1986), can be the only source of phenotypic variation for clonal populations (Molnár et al. 2015). From a molecular perspective, phenotypic plasticity is due to gene regulation (Leung et al. 2023). Gene expression is the expression of the genes of individuals with different or equal DNA sequences, mediated by the environment that can regulate (upregulate, downregulate or silence) the synthesis of functional products (proteins and functional RNA) at the cellular level. By controlling cell structure and function, genes play an important role in cellular differentiation, morphogenesis, and adaptability. Gene regulatory networks of phenotypic plasticity are largely unknown; however, models that integrate plant development regulatory networks with selection clearly demonstrate that plants can gradually acquire the ability to produce different phenotypes under different environmental conditions without sequence change (Pfennig and Ehrenreich 2014; Sakamoto and Innan 2024).

Epigenetic variation is a molecular modification that can alter gene regulation and function without changing the underlying DNA sequence (Duncan et al. 2014). These modifications include DNA methylation, histone modifications, and the action of non‐coding RNAs, all of which are triggered by the environment and influence the expression of genes involved in phenotypic traits and plastic responses (Huang et al. 2014). Most of the modifications are short‐lived and provide transient plasticity for local acclimation to fluctuating environments, while others can become transgenerational and result in inheritable adaptive phenotypic plastic traits (Duncan et al. 2014; Rajpal et al. 2022). Epigenetic variations may therefore play a crucial role in shaping functional traits in macrophytes (Chen et al. 2024).

Genome size is the total amount of DNA contained in a cell nucleus (Greilhuber et al. 2005), which is relatively constant for all individuals in a population, but it is known to vary widely between and within plant species over time, even on very short timescales (Trinder et al. 2025). Although numerous studies have explored the molecular mechanisms for genome size variation, which is primarily the expansion and contraction of repetitive DNA elements, the underlying processes of this change and the adaptive and evolutionary consequences remain unclear (Pellicer et al. 2018; Bhadra et al. 2023). Plant genome size constrains many growth‐related cellular processes, such as cell size, cell cycle duration, and metabolic rates (Morton et al. 2024), which in turn affect key functional traits, including seed mass, leaf structure, and stomatal size and density. Through these effects, genome size shapes critical aspects of a plant's life‐history strategy, including competitive ability, dispersal, and stress tolerance to environmental variables (Pellicer et al. 2018; Trinder et al. 2025).

This review aims to explore the multifaceted role of genetics, broadly including genomes and molecular variation in DNA sequence, in the expression of functional traits and reach a better understanding of the interplay between environment, genetics and functional traits in freshwater plants. We reviewed the available literature on wild macrophyte species that combined a functional trait‐based approach with environmental and genetic aspects. Here, we focus on vascular plants occurring in freshwater systems, spanning from lentic to lotic habitats and from greenhouse manipulated conditions to natural systems. By synthesizing the papers, we analyzed and discussed the role of genetic variation vs. environment in functional trait variation, the role of genetics in natural selection and phenotypic plasticity, and the effect of genetic drift vs. selection on traits. This review updates the state of the art on functional and genetic traits that have been investigated so far in macrophytes, as well as the experimental approaches that were followed by the researchers to disentangle the contribution of genetics to functional traits and advances the knowledge of plant adaptation processes in freshwater habitats.

## 2 Research Methods

Scopus main collection database was searched for the last time on 1st November 2025 using the three Boolean search strings including the TITLE‐ABS‐KEY fields. The first string focused on the “macrophyte” theme, so the Boolean terms used were: “aquatic plant” OR macrophyte OR hydrophyte OR helophyte OR pleustophyte OR “water plant” OR “wetland plant”; The second string focused on genetic aspects, so the Boolean terms used were: “genetic diversity” OR genetic OR “genetic variation” OR genome OR gene OR haplotype OR genomic OR genotype; finally, the third string focused on functional traits, so the Boolean terms used were: trait OR “functional trait” OR “plant trait”. Only articles published in English were considered. Initially, the research was conducted with no temporal restriction to get all available literature and not miss out on any publication. To have an overview of the completeness of our research, we conducted a preliminary search on Scopus database using the two search strings at the time, one for “macrophytes” AND “functional traits” and another for “macrophytes” AND “genetics”. The search for “macrophytes” AND “functional traits” returned 1318 articles, while the search combining “macrophytes” AND “genetics” resulted in 2010 articles. However, when all three search strings—“macrophytes”, “functional traits,” and “genetics”—were combined, the number of papers significantly decreased to only 169. To focus on recent advancements in genetics and current trends in the field of functional traits, we selected only articles published between 2012 and 2025. The number of articles per year increased considerably after 2012 compared to those between the first articles in 1994 and 2012. As a result, 136 papers were included in the analysis. A screening of the abstract and full text removed publications that did not fit with the aim of this review. For the articles to be included in the sample set, they had to include (i) freshwater macrophytes as the subject of the study; (ii) measurement of functional traits; (iii) genetic analysis or genetic information on the samples used; and (iv) environmental factors. We considered macrophytes (*sensu* Chambers et al. 2008), that is, photosynthetic organisms, large enough to be seen with the naked eye, whose life cycle depends entirely or partially on aquatic or periodically flooded environments. We focused exclusively on wild freshwater plant species and excluded cultivated plants such as cultivated rice ( *Oryza sativa* ). In terms of habitats, the focus was on natural and artificial freshwater habitats, including continental lentic and lotic ecosystems, such as lakes, ponds, wetlands, rivers and streams. We considered functional traits of the entire plants, encompassing both below and above‐ground traits. The TRY database (www.try‐db.org), an open‐access global repository of plant functional traits collected by researchers and integrated from pre‐existing databases (Kattge et al. 2020), was consulted to verify and validate whether the traits studied in the selected papers met the concept of “functional trait”, ensuring consistency in the comparison of standardized traits across the different studies. We also added the category of spectro‐functional traits, that are the ones collected with remote‐sensing techniques (Castellani, Coppi, et al. 2023). For genetic aspects we considered (i) direct genetic analysis, i.e., genetic information obtained with molecular or genomic markers (e.g., SSR, AFLP, SNPs, genome size); (ii) indirect genetic information obtained with quantitative genetics approaches from phenotypic variance partitioning into genetic and environmental components or (iii) indirect genetic information deduced from the experimental design (experiments with distinct genotypes, genotypes of distinct phyletic lineages, of distinct populations or of different species). Finally, we reviewed studies that examined plant responses to different types of abiotic and biotic factors at the sites where the samples were collected or under common or manipulated environmental conditions in controlled conditions (hereafter all included in “common garden experiments”). Overall, only 21 papers met the inclusion criteria and were included in the review.

To analyze the information collected from the papers, we prepared a database in which we recorded *Name of the species*, *Genetic groups* (species, lineages, haplotypes, populations, genotypes, clones), *Species number* (number of different species analyzed in the study), *Experimental design* (field study, common garden, field and common garden), *Functional traits* (*sensu* Violle et al. 2007) and *Genetic variation* traits (Data S1). Plant functional traits were divided into seven trait categories: *morphology* (related to measures of plant structure size); *tissue chemistry* (associated with the elemental composition of plant's tissue, such as C, N, P); *physiology* (related to plant processes, such as photosynthesis and respiration); *reproduction* (any trait related to vegetative or sexual reproduction, including number of flowers, number of seeds, seed size, vegetative propagule size); *growth* (biomass and growth rates); *herbivory* (plant traits that influence plant interactions with herbivores, such as leaf toughness and total phenolics content); *Spectro‐functional traits* (any trait sensed with remote sensing at plant level, like green leaf index (GLI) and mesophyll structure complexity). Genetic studies were very variable concerning the investigated genetic aspects that were related to the environmental conditions and the variation in phenotypic traits and were classified in *Genotypic variation* (referring to intraspecific genetic differences due to different genotypes, or to groups of genotypes with different phylogeographic or ecological background), *Genetic diversity* (amount of genetic variation detected with molecular markers (SSR, AFLP or SRAP) and quantified with specific indices like gene diversity (He), Shannon diversity index (I), or genetic differentiation (F_st_)), *Epigenetic variation* (any type of epigenetic trait due to DNA methylation), *Quantitative genetic variation* (traits obtained from phenotypic quantitative traits through heritability coefficient (H^2^), partitioning of phenotypic variance, Q_st_ index and Q_st_‐F_st_ comparison (Q_st_ indicates differentiation among populations in quantitative phenotypic traits and F_st_ genetic differentiation)), *Gene expression* (analysis of the expression of specific genes or of the entire transcriptome, detecting differences due to gene regulatory mechanisms in response to environmental conditions) and *Genome size* (c‐value, measuring the quantity of DNA in the nucleus of a cell). Finally, a Multiple Correspondence Analysis (MCA), suitable for large data matrices of multivariate nominal categorical variables, was performed with RStudio packages *FactoMineR* and *FactoExtra*. The results were then visualized using the customized 3D scatterplots created with the *Scatterplot3d* package.

## 3 Results and Discussion

### 3.1 Overview of the Examined Papers

The 21 reviewed papers included 15 different species with mixed (sexual and vegetative) reproductive strategies (Data S1). *Phragmites australis* was the most frequently studied species (reported in seven of the 21 papers), as it is an ideal model due to its extensive distribution, wide ecological range, high genetic diversity, and phenotypic plasticity.

Most studies were at the intraspecific level and compared genotypes, populations, and phylogenetic lineages (or haplotypes). Only a few studies compared two or more species to assess species response to biotic and abiotic factors (Hart et al. 2019; Al‐Dakhil et al. 2023; Castellani, Dalla Vecchia, et al. 2023; Jiang et al. 2025).

Twelve papers studied macrophytes grown under common garden conditions, using the different types of growth facilities (e.g., greenhouse, climate chamber and mesocosm). Field studies in lakes, rivers and marshland were the second most common type of research (Castellani, Coppi, et al. 2023; Chen et al. 2024; Dalla Vecchia et al. 2024; Aghaei et al. 2025), and a few studies combined field research with common garden (Zhou et al. 2014; Born and Michalski 2019; Jewell and Bell 2023; Wei et al. 2025).

**FIGURE 1:**
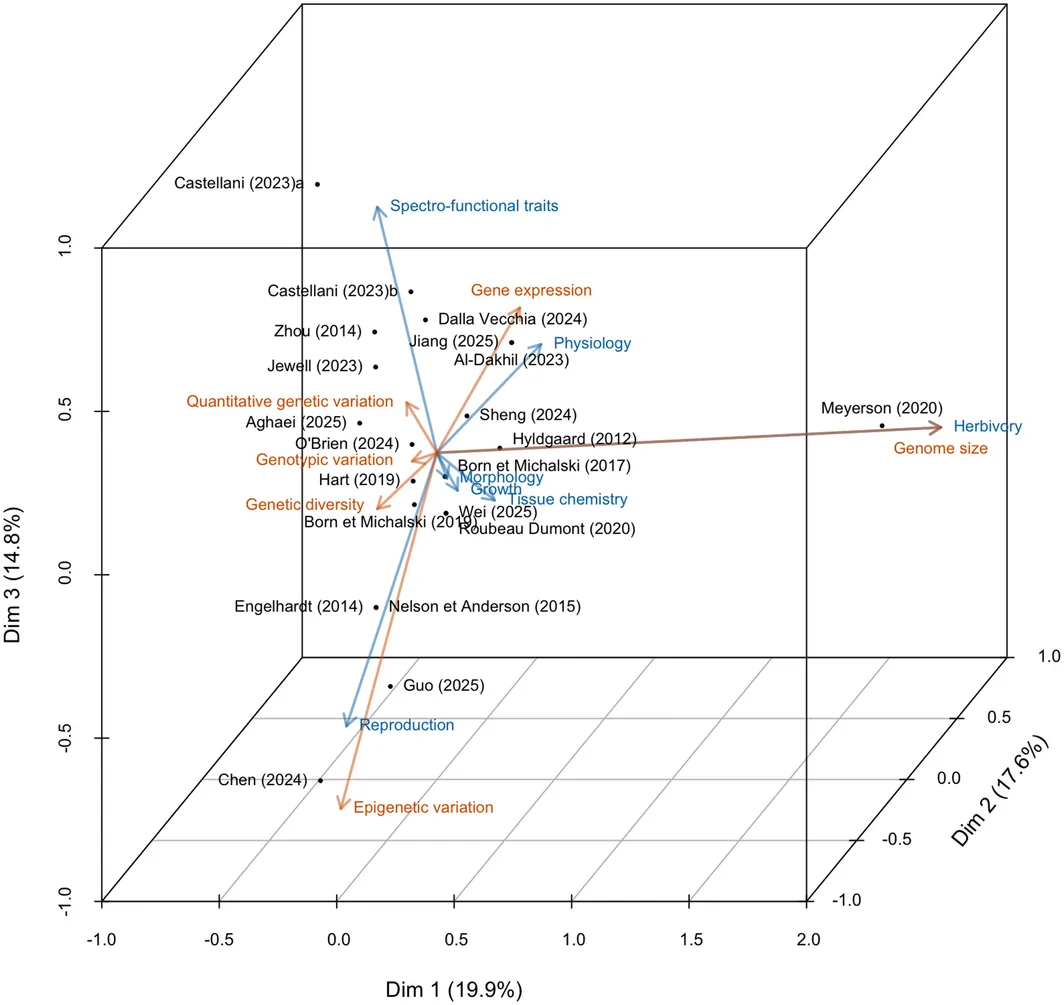
3D MCA scatterplot of the approaches used in the 21 papers included in this review. Each point represents a study (black dots), and arrows represent functional (blue) and genetic traits (yellow). The three axes correspond to the first three MCA dimensions (Dim 1–3), with the percentage of explained variance shown in parentheses. To interpret the graph, the length of the arrows indicates the traits that most differentiate the papers. Longer arrows correspond to a low number of studies that considered those traits, while shorter arrows indicate a larger number of studies.

The MCA provides an overview of the traits that were most or least studied in the reviewed papers and which genetic traits were studied for specific functional traits (Figure 1). Genetic and functional traits studied in a single or a few studies are those represented by long arrows, like *epigenetic variation, genome size, gene expression*, and *spectro‐functional traits*. Thus, papers located farthest from the center of the factorial space are those based on less common traits or unique combinations of genetic and functional traits. For example, Meyerson et al. (2020) is the only study addressing the effect of genome size on herbivory functional traits, while Jiang et al. (2025) and Al‐Dakhil et al. (2023) are the only papers that study gene expression in relation to physiology. The role of quantitative genetic variation in spectro‐functional traits is exclusive to Castellani, Dalla Vecchia, et al. (2023) and the study of epigenetic and genetic diversity on reproductive traits is exclusive to Chen et al. (2024) and Guo et al. (2025). In contrast, most papers focused on the role of genotypic variation on morphology and growth traits, which are represented by the shortest arrows in the MCA. The traits that were most frequently studied cluster together close to the center of the scatterplot.

**FIGURE 2:**
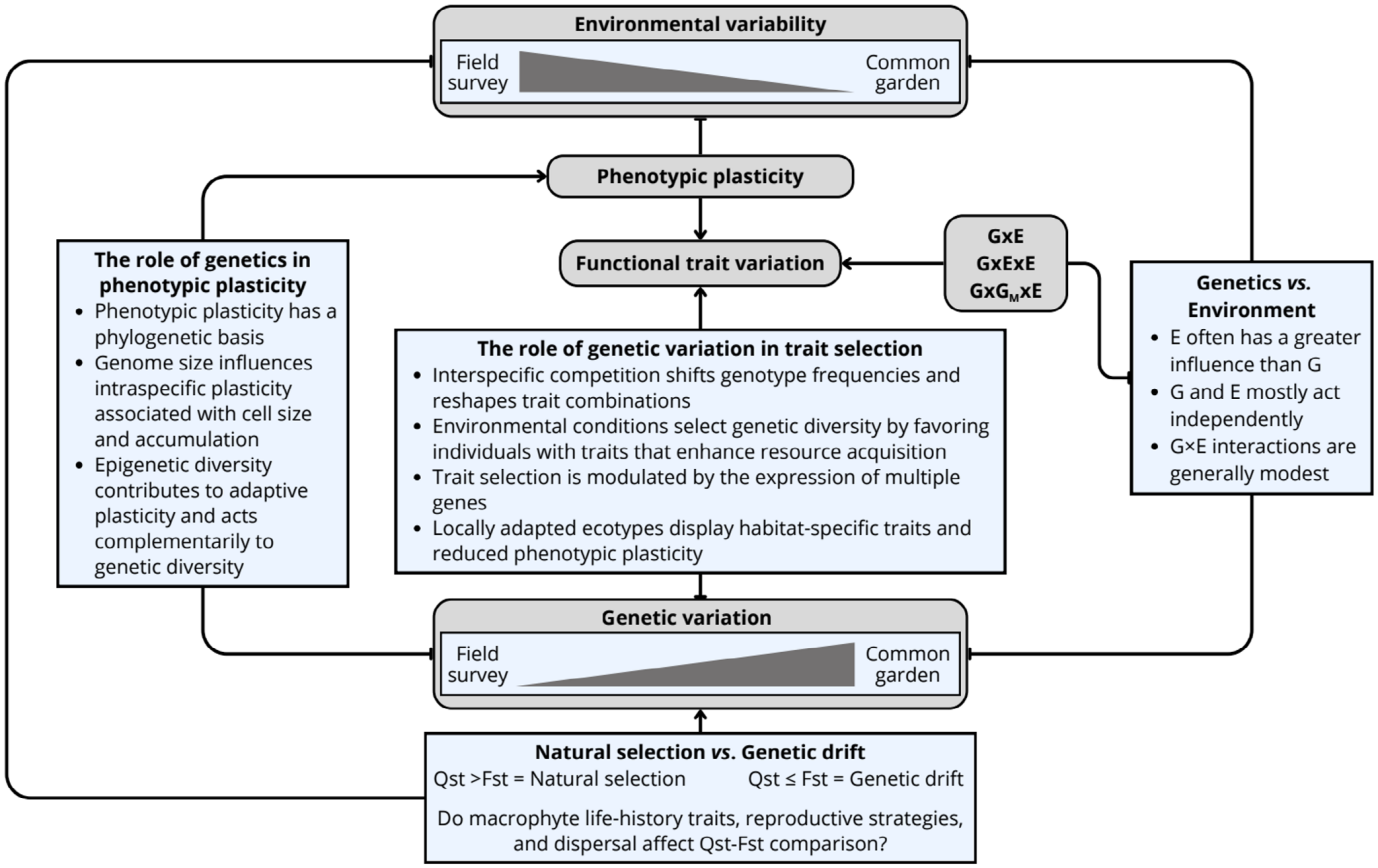
Conceptual model of the relationships between macrophyte functional traits and the mechanisms underlying their variation. The gray boxes represent the key processes driving trait variation: Environment variability, phenotypic plasticity, genetic variation and genotype‐environment interactions (G × E, G × E × E, G × Gm × E). The light blue boxes summarize the main concepts of the reviewed studies for each subsection of the “Results and discussion” section.

A conceptual map highlights and interconnects the multiple roles of genetic variation reviewed in the following chapters (Figure 2).

### 3.2 The Roles of Genetic Variation and Environment on Functional Traits

An effective approach to disentangle the relative contribution of genetics and environment to functional traits is through a common garden experiment. Phenotypic variation is due to a mixture of environmental and genetic sources, so transplanting the plants and growing them in a common garden, or exposing genetically distinct plants to manipulated common conditions, removes environmental variation, isolating the effect of genetic variation on traits (De Villemereuil et al. 2016). Functional trait variance can then be split between genetic and environmental variance (common garden vs. field variation or variance due to genotypes vs. imposed treatment). For instance, Jewell and Bell (2023) found that the phenotypic variation in leaf area and root length of *Lemna minor* genotypes across natural gradients of resources was primarily due to environment (over 70% of the variation within sites, and over 90% of the variation among sites) rather than to genetic differences assessed in common garden. Also, the genetic variance appeared higher within than among sites (Table 1).

**TABLE 1:** Partitioning of phenotypic variance in environmental (E), genetic (G), and genotype × environment interactions (G × E and G × G_M_ × E) components for each paper. G_M_ is microbial genetic composition. Percentages were derived via factorial ANOVA sum‐of‐squares (O'Brien et al. 2024), nested ANOVA (Jewell and Bell 2023), linear mixed‐model variance components (Castellani, Coppi, et al. 2023) or LDA inertia partitioning (Roubeau Dumont et al. 2020).

Author	Study type	Macrophyte species	Functional trait category	G%	E%	G × E%	G × G_M_%	G × G_M_ × E%	G_M_ × E%
O'Brien et al. 2024	Common garden	*Lemna minor*	Productivity	12–21	< 1	< 1	~4.6‐6.6	~4	< 1
			Morphology	6.6–10	< 1	< 1	~4.6‐6.6	3.2–4.1	< 1
Castellani, Coppi, et al. 2023	Field study	*Phragmites australis*	Spectro‐ functional traits	0–33.8	66.2–100				
Jewell and Bell 2023	Field and common garden	*Lemna minor*	Morphology	3.6‐26.3	73.7‐96.4				
Roubeau Dumont et al. 2020	Common garden	*Myriophyllum spicatum*	Morphology, productivity and tissue chemistry	18.8	14	10.9			

Alternative strategies based on field observations and genotyping of natural populations can provide reliable estimates in suitable study systems even without the use of common garden experiments. Castellani, Coppi, et al. (2023) found that most of the variation in *P. australis* spectral leaf traits, remotely sensed over large lake and marshland areas, was due to natural variable conditions (also in this case environmental variance ranged from 66% to 100%, depending on trait and ecological context) rather than to genetic background (Table 1). Interestingly, the genetic variance contribution of phylogenetic lineages was higher in aquatic than in terrestrial stands, probably, as suggested by the authors, because of stronger selective pressure in flooded environments than in land habitats, where phylogenetic variation was very low.

Instead, when plants were exposed to stress in common garden conditions, the role of genetic diversity appeared more important for trait variation than in wild conditions, perhaps because environmental heterogeneity is experimentally reduced, allowing genetically based differences to emerge more clearly. Under heavy metal stress, the partition of genetic variance exceeded that of the environment in the studies of Roubeau Dumont et al. (2020) and O'Brien et al. (2024) (Table 1). Under Cu stress, genetic variance among seven *Myriophyllum spicatum* genotypes collected from different rivers (G) accounted for 18% of the variation in functional traits associated with morphology, growth and tissue biochemistry, vs. 14% of variance due to Cu treatment (E) (Roubeau Dumont et al. 2020). G × E interaction accounted for 11% of the variation, indicating the important double role of genetics in shaping genotypes with different inherent Cu tolerance and adjusting differential gene expression in response to Cu exposure. A large part of the variation remained, however, unexplained, suggesting that other factors than genetics and environment had a role in shaping the studied functional traits. O'Brien et al. (2024) studied the effect of the microbiome (Gm variance) associated with genotypes of *L. minor* collected across an urban‐to‐rural ecotone on morphological and growth traits (G variance) when exposed to Zn (E variance) in common garden. Despite Zn exposure, G accounted for 6%–21% of the variation in *L. minor* frond traits versus less than 1% of variance due to Zn treatment and G × E interaction. The interaction G × Gm accounted for 4%–7% of the variation and G × Gm × E accounted for 3%–4%. Interestingly, Gm×E interaction accounted for less than 1% of the variation, revealing another important role of genetic variation in hosting genotype‐specific microbial communities, independent of the environment, at least in *L. minor* under Zn stress in common garden.

Most studies used ANOVA to assess the effects of genetic and environmental variance and their interaction on the variation in macrophyte traits, without explicitly partitioning their relative contributions (Data S2). Overall, the results of seven studies showed that genetic and environmental factors mostly acted independently, but never exclusively, on functional trait variation, while G × E interactions were generally weak. Significant environmental effects were more frequent than genetic effects for each functional trait category, with the exception of reproductive traits, which appeared to be more under genetic control. Still, the results depended on the experimental design. For example, in all three studies by Wei et al. (2025), Sheng et al. (2024), and Guo et al. (2025), functional trait variation in *P. australis* was more responsive to experimental salt treatments and/or climatic conditions than to lineage background, suggesting that *P. australis* primarily adapts through phenotypic plasticity. Instead, Zhou et al. (2014), using a broader latitudinal gradient and a reciprocal‐transplant design, found that genotypic diversity and G × E interactions explained a larger portion of the observed leaf nutrient variation of *Oryza rufipogon* populations than that explained by the environment alone.

In conclusion, with the limitations of a few and incomparable studies, the role of the environment appears stronger than that of genetic variation on functional trait variation of freshwater plants not associated with reproduction and in non‐stressful field conditions (Figure 2). Under stress imposed in common garden experiments, where environmental heterogeneity is reduced, the genetic contribution to trait variation increases. Interestingly, G × E interaction appeared less important than environment and genetic variation alone; however, the importance of this interaction depended on species, environmental conditions (including microbiotic interactions), and traits. Further research is needed to confirm whether these emerging patterns are robust and generalizable across freshwater plant systems.

### 3.3 The Role of Genetic Variation in Trait Selection

Competition is a fundamental driver in the process of natural selection for species to adapt and evolve in response to their environment. How competition affects trait selection and genetic variation was shown by the experiment of Hart et al. (2019) in which *Lemna minor* and *Spirodela polyrhiza* were grown together in experimental ponds and competed for the same resources. The interspecific competition caused a rapid change in the genotypic frequencies of *L. minor* , with a few genotypes becoming more frequent than at the beginning of the experiment and others decreasing in frequency, but not of *S. polyrhiza* . At the end of the experiment, changes in genotype frequencies of *L. minor* resulted in a higher specific leaf area (SLA) and root length/dry mass ratio and a lower leaf dry matter content (LDMC), suggesting a shift toward a more resource‐acquisitive strategy. This led to an increased growth rate, but also to a higher sensibility to competition, balancing the competitiveness of the two species without increasing niche differentiation as could be expected from the competition between sympatric species (Hart et al. 2019). The study of Dalla Vecchia et al. (2024) measured the correlation between genetic diversity and environmental variables in different natural populations of *Nuphar lutea* and found that water electrical conductivity was negatively correlated with the genetic diversity of the populations, suggesting that specific environmental conditions selected distinct genotypes over time. Similarly to the experiment with *L. minor* , acquisitive behavior resulted successful also for *N. lutea* genotypes, as SLA increased while LDMC and leaf structural complexity decreased in the genotypes that survived higher conductivity. Acquisitive traits, however, do not account for the competitive advantage of all freshwater plants. In the Chinese populations of the emergent *P. australis* , Chen et al. (2024) found that genetic diversity was positively correlated with soil conductivity and negatively correlated with temperature, precipitation and nutrient conditions, indicating that in harsher conditions (low temperature, low precipitation, low nutrient and high salinity), hence under stronger selective pressure, *P. australis* genetic diversity was highest. In this study, genetic diversity was correlated with variation in reproductive traits (measured as flower and rhizome biomass), suggesting that under harsh conditions, where genetic diversity is highest, this macrophyte can increase variation in reproductive traits, rather than nutrient acquisition, as a conservative strategy ensuring survival and adaptive capacity. In addition to inherent genetic variation due to sequence variation, natural selection also acts on gene regulation and expression. Regulatory transcription factors are particularly sensitive to environmental influences, which modulate the spatio‐temporal and quantitative patterns of gene expression. This plasticity in gene‐expression regulation generates phenotypic variation, providing the raw material on which selection can act.

With a transcriptomic approach, Jiang et al. (2025) compared cold tolerance in *Juncus articulatus* and *Paspalum vaginatum* , two sympatric species collected from coastal estuarine wetlands in the south of China. Although cold impaired physiological traits in both species, *J. articulatus* maintained better performance than *P. vaginatum* . Transcriptomic analysis identified more than 62,000 genes differentially expressed in the two species under cold treatment, many of which were involved in the regulation of key metabolic pathways (glutathione, starch, sucrose, and flavonoid biosynthesis) that increased antioxidant defenses and the accumulation of soluble sugars in *J. articulatus* , conferring a competitive advantage under low‐temperature stress.

In contrast, the seven genes examined by Al‐Dakhil et al. (2023) are involved in specific different physiological pathways related to salinity tolerance and many of them are highly conserved across species because they perform essential cellular functions and are structurally constrained. Consequently, they represent an evolutionarily stable portion of the broader gene‐expression machinery. Under salt stress, the authors observed a rapid induction of regulatory transcription factors, followed by activation of ion‐transport genes to counter Na^+^ accumulation in the cells and restore ion homeostasis. Together, these two studies highlight that the effect of environmental variation on phenotypic variation involves several genes and is not straightforward because genes do not respond to the environment in a simple, linear way. Environmental signals influence transcription factors, chromatin accessibility, and epigenetic alterations, producing context‐dependent patterns of gene expression rather than uniform responses resulting in highly context‐dependent phenotypes. This complexity makes gene–environment interactions difficult to predict and central to understanding phenotypic variation and plasticity.

Evidence of adaptation was provided by Nelson and Anderson (2015) by showing that *Phalaris arundinacea* genotypes originating from upland habitats grew taller and had longer panicles when grown in dry soil conditions that resembled their habitat of origin than when grown in moist soils. Similarly, genotypes of *Vallisneria americana* from sheltered sandy habitats had longer rhizomes and higher turion production when grown in conditions that resembled their habitat of origin and performed more poorly in gravel sediments (Engelhardt et al. 2014). Interestingly, this pattern was not symmetric, as *P. arundinacea* genotypes collected from wetland habitats had no substantial trait changes when grown in the two contrasting environments and did not differ from the upland genotypes when grown in dry soils. Likewise, *V. americana* genotypes from gravel sediments didn't exhibit a home‐site advantage, but benefited from exposure to the more favorable sandy sediments and some genotypes even exceeded plant size and turion production of sandy genotypes. These findings suggest that local adaptation is not necessarily expressed as a reciprocal home‐field advantage across contrasting habitats, as variation in habitat quality can become an overriding factor that affects genotypes' performance, making local adaptation more difficult to detect in phenotypic responses.

### 3.4 The Role of Genetics in Phenotypic Plasticity

Macrophyte invasions provide good models to study adaptation processes in a new range. The study of Hyldgaard et al. (2012) measured differences in phenotypic plasticity between invasive (New Zealand) and native (Denmark) *Ceratophyllum demersum* populations by quantifying trait variation in response to N water availability. In their common garden experiment, the introduced population had higher phenotypic plasticity than the native populations in several traits associated with fast growth (relative growth rate, stem length, tissue N content, SLA, photosynthetic rates). This suggests the genetic or phylogenetic background of the populations influences their plastic responses, supporting the idea that phenotypic plasticity can have a genetic basis that enhances plant fitness, either dependently or independently of environmental conditions. In contrast, Meyerson et al. (2020) found no evidence that the invasive lineage of *P. australis* had higher trait plasticity than the native lineage. Nevertheless, their common garden study revealed that genome size, rather than phylogenetic background, influenced the phenotypic plasticity of different traits in native and invasive lineages differently, under saline and climatic treatments, providing further support that phenotypic plasticity has a genetic basis. For the invasive lineage, plasticity in stomatal conductance and aboveground phenolics decreased, while plasticity in belowground percent C and N increased with increasing genome size. For the native lineage, the relationship was positive for aboveground C content and negative for leaf toughness. Differences in genome size can therefore have a strong effect on the competitive ability of the two distinct phylogenetic lineages. Plasticity in stomatal conductance affects water use and photosynthetic efficiency, plasticity in above‐ and below‐ground resource allocation can affect survival capacity, whereas plasticity in phenolics and leaf toughness can affect species interactions and the capacity to defend from herbivores.

Epigenetic diversity also plays a role in shaping the phenotypic plasticity of functional traits, as explored by Chen et al. (2024) with a field study of *P. australis* populations. Genetic sequences and epigenetic features were found to act jointly in regulating gene trait expression, although these two sources of variation influenced the plasticity of different traits. Genetic variation was related to the coefficient of variation of reproductive traits (spike and rhizome biomass), whereas epigenetic variation was related to the coefficient of variation of growth traits (plant height, stem and leaf biomass). Consistent with Chen et al. (2024), a subsequent common garden study by Guo et al. (2025) of two *P. australis* phylogenetic lineages, each including genotypes of different provenances, found a strong positive correlation between epigenetic and genetic variation within lineage. As in the previous study, genetic and epigenetic variation were associated with different traits, but in this case, epigenetic effects on trait variation were weaker, likely reflecting the uniformity of common‐garden conditions. Genetic and epigenetic variation do not necessarily align because epigenetic modifications adjust quickly to environmental conditions, while genetic variation changes through slower evolutionary processes. As a result, geographic epigenetic patterns do not necessarily mirror genetic ones in macrophyte populations: Chen et al. (2024) reported marked latitudinal variation in genetic diversity, while epigenetic diversity did not vary consistently and remained uniformly high across populations. Opposite patterns were described for invasive *Alternanthera philoxeroides* and *Arundo donax* that have geographic epigenetic structure in their introduced ranges, despite the populations having no genetic diversity being the invasions caused by one single genotype (Guarino et al. 2019; Li et al. 2024). Taken together, the evidence indicates that phenotypic plasticity is shaped by multiple genomic layers (phylogenetic identity, genome size and environmentally responsive epigenetic variation) whose interactions are complex and not yet fully understood. Further research is therefore warranted to clarify how these genomic traits interplay and jointly shape plastic responses.

### 3.5 Genetic Drift vs. Natural Selection

Alongside the environmental filter posed by natural selection, genetic and phenotypic variation of macrophytes can be shaped by other evolutionary and demographic processes. Genetic drift in small and isolated populations and intraspecific variation in reproductive strategies may significantly contribute to shaping and distributing genetic diversity within and among macrophyte populations, affecting the interplay of genetics and environment independently of selection.

The quantitative comparison of phenotypic differentiation (Q_st_) and genetic differentiation (F_st_) (obtained by the analysis of variance) can be used to estimate the relative contributions of genetic drift and selection to phenotypic variation among populations. When Q_st_ exceeds F_st_, it indicates (environmental‐driven) selection and adaptive traits selection; when, instead, Q_st_ is lower than F_st_, it is interpreted as the effect of neutral divergence due to genetic drift. With this approach, Born and Michalski (2019) found that genetic differentiation (F_st_) was higher than trait differentiation (Q_st_) in response to N supply among populations of *J. effusus* , suggesting that the observed variation in several growth and biochemical traits was due to genetic drift among populations. In this case, however, the high F_st_ values among populations were largely a consequence of self‐pollination and high inbreeding within populations, meaning that the observed trait variation reflected the close relatedness of genotypes rather than divergent responses to N availability among populations. In *P. australis* wild populations the Q_st_‐F_st_ comparison revealed directional selection of morphological, growth and physiological traits in most of the studied populations, as trait differentiation (P_st_ in this study, equivalent to Q_st_) consistently exceeded genetic differentiation (F_st_) among populations for almost every studied trait (Castellani, Dalla Vecchia, et al. 2023). On the contrary, *N. lutea* populations from the same sites showed a more complex P_st_‐F_st_ pattern depending on population and functional trait, with P_st_ values consistently lower or similar to F_st_ values for all traits. According to this approach, differences in functional traits among populations were better explained by genetic drift than by trait selection, like for *J. effusus* . Unlike *P. australis*, which is dispersed by wind, hydrology can have a strong impact on *N. lutea* seeds and vegetative dispersal, hence on the distribution of genetic diversity and the effect of genetic drift (Castellani, Dalla Vecchia, et al. 2023).

Because of the large variation in life history traits among freshwater plant species, especially in breeding and dispersal abilities, the Q_st_‐F_st_ approach of quantitative genetics might not always be the best method to apply to recognize adaptive vs. non‐adaptive traits in macrophytes. The high frequency of selfing and clonality in freshwater plants may in fact hide the signature of selection in quantitative traits (Q_st_) (as pointed out by Born and Michalski (2019) for *J. effusus* ) by overestimating the F_st_ index of genetic differentiation (Lambertini et al. 2010). Selection could therefore have a stronger role in freshwater environments than assessed in these studies. Ongoing research is producing new concepts and metrics of genetic variance partitioning among populations, accounting for clonality and mixed mating systems that could better capture genetic differentiation among macrophyte populations than classical F_st_ metrics (Jost 2008; Chao et al. 2015; Kosman et al. 2024).

## 4 Broader Context and Forward Outlook

When referring to variation in functional traits, adaptation due to natural selection is often regarded as the main process that shapes this variation. However, other processes such as genetic drift and phenotypic plasticity are also important and cannot be assessed without a genetic study alongside the study of functional traits and environment. In addition, genetic variation can affect functional trait expressions in many different ways. It is not only genotypic variation due to DNA sequence variation among individuals that interacts with the environment, but also gene regulation and expression, DNA methylation and genome size, as seen in this review, and the phenotype is the result of the interaction of all these factors. Other genetic mechanisms for which we did not find relevant papers for the review are polyploidy (i.e., the heritable condition of possessing more than two complete sets of chromosomes) and interspecific hybridization that can affect phylogeny, genome size and regulation, as well as population dynamics, genetic diversity and adaptive trait evolution. For example, Grewell et al. (2016) demonstrated that diploid and decaploid congeners of *Ludwigia* responded differently to nutrient availability, with the diploid having higher growth rate and biomass accumulation than the polyploid. Gallego‐Tévar et al. (2019) showed that the hybrid *Spartina densiflora* × *foliosa* exhibited greater tolerance to salinity and tidal inundation than its parental species, because of the effects of transgressive hybridization in key functional traits. In addition, genetic traits, like functional traits and environment, are dynamic. Gene regulation modulated by the environment is very context‐dependent and life history traits (mating system and dispersal ecology) as well as phylogenetic relationships, amount and distribution of genetic diversity further shape how phenotypes respond to the environment, whether selection is acting or not.

With this work, we presented an overview of the multifaceted role of genetics in shaping functional traits and highlighted the critical importance of integrating functional traits with genetic variation and environmental factors to achieve a more comprehensive understanding of freshwater macrophytes' responses, adaptation, and eco‐evolutionary processes expressed in traits. Over the last 15 years, there has been a significant increase in publications integrating genetics in the study of macrophyte functional traits, with multiple research strategies and their pros and cons. However, the number of studies is still limited.

Recent reviews from marine literature have increasingly emphasized the need to integrate ecological and genetic perspectives also into marine macrophyte studies, that, like freshwater plants, are much less investigated than terrestrial plants concerning the mechanisms underlying adaptation and evolution (King et al. 2018). Marine macrophytes inhabit highly dynamic coastal environments that are vulnerable to multiple natural and anthropogenic disturbances, including sea level rise, ocean warming and acidification (Nguyen et al. 2020). These rapid environmental changes strongly influence plant performance, traits and resilience, making marine macrophytes informative systems for the study of adaptation processes (Smale and King 2024). Duarte et al. (2018) reviewed the complementary roles of genetic diversity, epigenetic mechanisms and the associated microbiome in generating phenotypic variation in seagrass meadows and macroalgal forests in response to climate change effects. All three factors were shown to create phenotypic variation that may either enhance fitness within individuals (plasticity) or be subject to selection and ultimately, adaptation (Duarte et al. 2018). In the clonal seagrass meadows of *Zostera marina* , Jueterbock et al. (2020) demonstrated that DNA methylation variation can be independent from the underlying genetic variation and rather be associated with environmentally induced responses. Interestingly, they found that DNA methylation patterns varied among shoots in functionally relevant genes involved in photosynthesis and in protein damage repair and prevention, linking epigenetics to gene expression, and suggesting that in clonal aquatic plants epigenetic variation may play a similar role to genetic variation in providing resilience and population stability. The conundrum of disentangling phenotypic plasticity from selection and local adaptation in seagrass and macroalgae was also addressed by King et al. (2018) and Hu et al. (2020), both of which discuss the potential of combining ecological experimental approaches (e.g., common garden and reciprocal transplant experiments) with modern DNA‐based technologies (e.g., NGS, genome scans and SNP genotyping) to link genetically based trait differentiation to specific adaptive loci. Together, these approaches can help understand whether population‐level differences are genetically fixed or environmentally induced and identify specific loci associated with divergent selection across habitats, thereby helping close the loop between genetic variation, environmental context, and functional trait expression. Omics technologies, including genomics, epigenomics, transcriptomics and metabolomics are still under exploited in marine as well as in freshwater macrophytes (Zhang et al. 2024), but are essential to study adaptation processes as genome‐wide analyses can detect genetic variation and spot genes, regulatory pathways, and metabolic networks associated with phenotypic variation and plasticity, capturing the full spectrum of genome‐wide responses to environmental variation (Zaidem et al. 2019).

Other gaps that emerge from our review are the study of a few model macrophytes (duckweeds and *P. australis* were the most studied freshwater plants), the focus on genotypic variation among individuals as the main source of trait variation and on few functional traits (mostly related to morphology and growth). In addition, the studies were mostly carried out in common gardens where few abiotic factors could be manipulated. Therefore, there is a need for more extensive, multi‐factorial and longer‐term investigations that would better capture macrophytes' responses also under natural conditions, present and future scenarios (Bolpagni 2021). Certainly, the integration of remote sensing technologies with genetic diversity can be very powerful when studying natural populations, since it enables large‐scale and multi‐temporal monitoring of trait variation in relation to the micro‐ and macro‐ecological and evolutionary dynamics at fine and large scale, overcoming the limitations of traditional field‐based and common garden methods, which are spatially and ecologically restricted (Castellani, Coppi, et al. 2023). In addition, the current advancement of artificial intelligence (AI) technologies can automate large‐scale remote sensing data collection and analysis, assist species identification and provide data that can track community changes and predict future ecological trends (Zhang et al. 2024). The integration of genetic information in such tools is fundamental to understanding the evolutionary significance of the changes that are detected. AI could be the tool to link these data; however, dedicated algorithms should be developed first to account for the complex relationships between genetic, environmental and phenotypic data. This field still appears to be in its early stages for macrophyte research.

Despite the variation and the limitations of the methods applied in the selected papers, it seems clear that the phenotypic expression of macrophytes is the result of the additive and interactive roles of genetics and environment, including also epigenetics in genetic factors, and the study of both factors prevents bias and misinterpretation. Overall, understanding the mechanisms that shape the phenotypic expression provides a more holistic understanding of the implications of trait variation and a realistic assessment of the ecological and evolutionary dynamics of macrophytes. Such knowledge is essential for biodiversity conservation and habitat restoration, for the selection of resilient and locally adapted species and genotypes, as well as for conserving genetic diversity and processes that can increase the adaptive potential of native populations to the current challenges and prevent the negative ecological impacts of invasive species.

## Author Contributions

**Beatrice Fois:** conceptualization (equal), data curation (equal), formal analysis (equal), investigation (equal), validation (equal), writing – original draft (equal), writing – review and editing (equal). **Alice Dalla Vecchia:** validation (equal), writing – review and editing (equal). **Giovanni Campiello:** data curation (equal), investigation (equal), validation (equal), writing – review and editing (equal). **Rossano Bolpagni:** conceptualization (equal), data curation (equal), investigation (equal), validation (equal), writing – review and editing (equal). **Carla Lambertini:** conceptualization (equal), data curation (equal), formal analysis (equal), investigation (equal), validation (equal), writing – original draft (equal), writing – review and editing (equal).

## Conflicts of Interest

The authors declare no conflicts of interest.

## Supporting information


**Data S1:** Database of collected data for each selected paper.

**Data S2:** Results of the ANOVA‐family tests (two‐way or three‐way ANOVA, ANOVA within mixed‐effect models, or ANCOVA where covariates were included) assessing the significance of environment (E), genetics (G) and their interactions (G × E and G × E × E) effects on functional trait category for each paper. Significance was assessed via F‐test (or equivalent Chi‐square/Ancova tests) (*p < 0.05; **p < 0.01; ***p < 0.001; ns = not significant).

## Data Availability

All data supporting the findings of this review are derived from previously published studies, which are cited within the manuscript and Supporting information.

## References

[ece374326-bib-0001] Aghaei, N. , S. Afsharzadeh , and S. Abbasi . 2025. “Investigating of Intraspecific Diversity of *Potamogeton nodosus* Poir. In Iran Using Morphological Traits and Molecular Markers.” Iran Journal of Science 49, no. 2: 297–306. 10.1007/s40995-024-01733-6.

[ece374326-bib-0002] Albert, C. H. , F. Grassein , F. M. Schurr , G. Vieilledent , and C. Violle . 2011. “When and How Should Intraspecific Variability Be Considered in Trait‐Based Plant Ecology?” Perspectives in Plant Ecology, Evolution and Systematics 13, no. 3: 217–225. 10.1016/j.ppees.2011.04.003.

[ece374326-bib-0003] Al‐Dakhil, M. , W. Ben Romdhane , S. Alghamdi , and A. A. M. Ali . 2023. “Differential Morpho‐Physiological and Biochemical Responses of Duckweed Clones From Saudi Arabia to Salinity.” Plants 12, no. 18: 3206. 10.3390/plants12183206. 37765370 PMC10537559

[ece374326-bib-0004] Barrett‐Lennard, G. T. 1993. “The Phases and Focus of Empathy.” British Journal of Medical Psychology 66: 3–14. 10.1111/j.2044-8341.1993.tb01722.x. 8485075

[ece374326-bib-0005] Benelli, S. , and M. Bartoli . 2021. “Worms and Submersed Macrophytes Reduce Methane Release and Increase Nutrient Removal in Organic Sediments.” Limnology and Oceanography Letters 6, no. 6: 329–338. 10.1002/lol2.10207.

[ece374326-bib-0006] Bhadra, S. , I. J. Leitch , and R. E. Onstein . 2023. “From Genome Size to Trait Evolution During Angiosperm Radiation.” Trends in Genetics 39, no. 10: 728–735. 10.1016/j.tig.2023.07.006. 37582671

[ece374326-bib-0007] Bolpagni, R. 2021. “Towards Global Dominance of Invasive Alien Plants in Freshwater Ecosystems: The Dawn of the Exocene?” Hydrobiologia 848: 2259–2279. 10.1007/s10750-020-04490-w.

[ece374326-bib-0008] Born, J. , and S. G. Michalski . 2017. “Strong Divergence in Quantitative Traits and Plastic Behavior in Response to Nitrogen Availability Among Provenances of a Common Wetland Plant.” Aquatic Botany 136: 138–145. 10.1016/j.aquabot.2016.10.002.

[ece374326-bib-0009] Born, J. , and S. G. Michalski . 2019. “Trait Expression and Signatures of Adaptation in Response to Nitrogen Addition in the Common Wetland Plant *Juncus effusus* .” PLoS One 14, no. 1: e0209886. 10.1371/journal.pone.0209886. 30608976 PMC6319709

[ece374326-bib-0010] Bornette, G. , and S. Puijalon . 2011. “Response of Aquatic Plants to Abiotic Factors: A Review.” Aquatic Sciences 73: 1–14. 10.1007/s00027-010-0162-7.

[ece374326-bib-0011] Cao, J. J. , Y. Wang , and Z. L. Zhu . 2012. “Growth Response of the Submerged Macrophyte *Myriophyllum spicatum* to Sediment Nutrient Levels and Water‐Level Fluctuations.” Aquatic Biology 17, no. 3: 295–303. 10.3354/ab00484.

[ece374326-bib-0012] Castellani, M. B. , A. Coppi , R. Bolpagni , et al. 2023. “Assessing the Haplotype and Spectro‐Functional Traits Interactions to Explore the Intraspecific Diversity of Common Reed in Central Italy.” Hydrobiologia 850, no. 4: 775–791. 10.1007/s10750-022-05124-z.

[ece374326-bib-0013] Castellani, M. B. , A. Dalla Vecchia , R. Bolpagni , et al. 2023. “Genetic Drift Versus Natural Selection Affecting the Evolution of Spectral and Functional Traits of Two Key Macrophytes: *Phragmites australis* and *Nuphar lutea* .” Freshwater Biology 68: 1739–1750. 10.1101/2023.06.19.545543.

[ece374326-bib-0014] Chambers, P. A. , P. Lacoul , K. J. Murphy , and S. M. Thomaz . 2008. “Global Diversity of Aquatic Macrophytes in Freshwater.” Hydrobiologia 595, no. 1: 9–26. 10.1007/s10750-007-9154-6.

[ece374326-bib-0015] Chao, A. , L. Jost , T. C. Hsieh , K. H. Ma , W. B. Sherwin , and L. A. Rollins . 2015. “Expected Shannon Entropy and Shannon Differentiation Between Subpopulations for Neutral Genes Under the Finite Island Model.” PLoS One 10, no. 6: e0125471. 10.1371/journal.pone.0125471. 26067448 PMC4465833

[ece374326-bib-0016] Chelli, S. , M. Marignani , E. Barni , et al. 2019. “Plant‐Environment Through a Functional Traits Perspective: A Review of Italian Studies.” Plant Biosystems ‐ An International Journal Dealing with all Aspects of Plant Biology 153, no. 6: 853–869. 10.1080/11263504.2018.1559250.

[ece374326-bib-0017] Chen, Y. , J. Meng , C. Wang , T. Fang , Z. Jia , and F. Luo . 2024. “Correlations Among Genetic, Epigenetic, and Phenotypic Variation of *Phragmites australis* Along Latitudes.” Diversity and Distributions 30, no. 9: e13907. 10.1111/ddi.13907.

[ece374326-bib-0018] Dalla Vecchia, A. , and R. Bolpagni . 2022. “The Importance of Being Petioled: Leaf Traits and Resource‐Use Strategies in *Nuphar lutea* .” Hydrobiologia 849, no. 17–18: 3801–3812. 10.1007/s10750-022-04803-1.

[ece374326-bib-0019] Dalla Vecchia, A. , A. Coppi , M. Beatrice Castellani , et al. 2024. “Multidimensional Trait Variability in a Widespread, Paleoarctic Macrophyte: Functional, Spectral and Genetic Drivers.” Oikos 2024, no. 2: e10047. 10.1111/oik.10047.

[ece374326-bib-0020] Dalla Vecchia, A. , P. Villa , and R. Bolpagni . 2020. “Functional Traits in Macrophyte Studies: Current Trends and Future Research Agenda.” Aquatic Botany 167: 103290. 10.1016/j.aquabot.2020.103290.

[ece374326-bib-0021] De Villemereuil, P. , O. E. Gaggiotti , M. Mouterde , and I. Till‐Bottraud . 2016. “Common Garden Experiments in the Genomic Era: New Perspectives and Opportunities.” Heredity 116, no. 3: 249–254. 10.1038/hdy.2015.93. 26486610 PMC4806574

[ece374326-bib-0022] Diaz, S. , M. Cabido , and F. Casanoves . 1998. “Plant Functional Traits and Environmental Filters at a Regional Scale.” Journal of Vegetation Science 9: 113–122. 10.2307/3237229.

[ece374326-bib-0023] Diaz, S. , J. G. Hodgson , K. Thompson , et al. 2004. “The Plant Traits That Drive Ecosystems: Evidence From Three Continents.” Journal of Vegetation Science 15, no. 3: 295–304. 10.1111/j.1654-1103.2004.tb02266.x.

[ece374326-bib-0024] Duarte, B. , I. Martins , R. Rosa , et al. 2018. “Climate Change Impacts on Seagrass Meadows and Macroalgal Forests: An Integrative Perspective on Acclimation and Adaptation Potential.” Frontiers in Marine Science 5: 190. 10.3389/fmars.2018.00190.

[ece374326-bib-0025] Duncan, E. J. , P. D. Gluckman , and P. K. Dearden . 2014. “Epigenetics, Plasticity, and Evolution: How Do We Link Epigenetic Change to Phenotype?” Journal of Experimental Zoology Part B: Molecular and Developmental Evolution 322, no. 4: 208–220. 10.1002/jez.b.22571. 24719220

[ece374326-bib-0026] Engelhardt, K. A. M. , M. W. Lloyd , and M. C. Neel . 2014. “Effects of Genetic Diversity on Conservation and Restoration Potential at Individual, Population, and Regional Scales.” Biological Conservation 179: 6–16. 10.1016/j.biocon.2014.08.011.

[ece374326-bib-0027] Engloner, A. I. , K. Németh , P. B. Kós , E. Meglécz , and J. Bereczki . 2023. “Genetic Diversity of the Submerged Macrophyte *Ceratophyllum demersum* Depends on Habitat Hydrology and Habitat Fragmentation.” Frontiers in Plant Science 14: 1277916. 10.3389/fpls.2023.1277916. 38023870 PMC10665863

[ece374326-bib-0028] Gallego‐Tévar, B. , B. J. Grewell , R. E. Drenovsky , and J. M. Castillo . 2019. “Transgressivity in Key Functional Traits Rather Than Phenotypic Plasticity Promotes Stress Tolerance in a Hybrid Cordgrass.” Plants 8, no. 12: 594. 10.3390/plants8120594. 31842356 PMC6963473

[ece374326-bib-0029] Greilhuber, J. , J. Doležel , M. A. Lysák , and M. D. Bennett . 2005. “The Origin, Evolution and Proposed Stabilization of the Terms “Genome Size” and “C‐Value” to Describe Nuclear DNA Contents.” Annals of Botany 95, no. 1: 255–260. 10.1093/aob/mci019. 15596473 PMC4246724

[ece374326-bib-0030] Grewell, B. J. , M. J. Skaer Thomason , C. J. Futrell , M. Iannucci , and R. E. Drenovsky . 2016. “Trait Responses of Invasive Aquatic Macrophyte Congeners: Colonizing Diploid Outperforms Polyploid.” AoB Plants 8: plw014. 10.1093/aobpla/plw014. 26921139 PMC4823780

[ece374326-bib-0031] Guarino, F. , A. Cicatelli , G. Brundu , G. Improta , M. Triassi , and S. Castiglione . 2019. “The Use of MSAP Reveals Epigenetic Diversity of the Invasive Clonal Populations of *Arundo donax* L.” PLoS One 14, no. 4: e0215096. 10.1371/journal.pone.0215096. 30964932 PMC6456200

[ece374326-bib-0032] Guo, X. , H. Song , P. Wu , et al. 2025. “Intraspecific Elementome Variation of the Clonal Grass *Phragmites australis* Reflects Environmental Variation More Than Genetic and Epigenetic Variation.” Journal of Plant Ecology 18, no. 4: rtaf070. 10.1093/jpe/rtaf070.

[ece374326-bib-0033] Hart, S. P. , M. M. Turcotte , and J. M. Levine . 2019. “Effects of Rapid Evolution on Species Coexistence.” Proceedings of the National Academy of Sciences of the United States of America 116, no. 6: 2112–2117. 10.1073/pnas.1816298116. 30659157 PMC6369787

[ece374326-bib-0034] Hu, Z.‐M. , K.‐L. Zhong , F. Weinberger , D.‐L. Duan , S. G. A. Draisma , and E. A. Serrão . 2020. “Linking Ecology to Genetics to Better Understand Adaptation and Evolution: A Review in Marine Macrophytes.” Frontiers in Marine Science 7: 545102. 10.3389/fmars.2020.545102.

[ece374326-bib-0035] Huang, C.‐L. , J.‐H. Chen , M.‐H. Tsang , J.‐D. Chung , C.‐T. Chang , and S.‐Y. Hwang . 2014. “Influences of Environmental and Spatial Factors on Genetic and Epigenetic Variations in Rhododendron Oldhamii (Ericaceae).” Tree Genetics & Genomes 11, no. 1: 823. 10.1007/s11295-014-0823-0.

[ece374326-bib-0036] Huotari, T. , and H. Korpelainen . 2013. “Comparative Analyses of Plastid Sequences Between Native and Introduced Populations of Aquatic Weeds Elodea Canadensis and *E. nuttallii* .” PLoS One 8, no. 4: e58073. 10.1371/journal.pone.0058073. 23620722 PMC3631202

[ece374326-bib-0037] Hyldgaard, B. , B. Sorrell , B. Olesen , T. Riis , and H. Brix . 2012. “Geographically Distinct *Ceratophyllum demersum* Populations Differ in Growth, Photosynthetic Responses and Phenotypic Plasticity to Nitrogen Availability.” Functional Plant Biology 39, no. 9: 774–783. 10.1071/FP12068. 32480828

[ece374326-bib-0038] Islam, T. , M. Hamid , I. A. Nawchoo , and A. A. Khuroo . 2024. “Leaf Functional Traits Vary Among Growth Forms and Vegetation Zones in the Himalaya.” Science of the Total Environment 906: 167274. 10.1016/j.scitotenv.2023.167274. 37741392

[ece374326-bib-0039] Jewell, M. D. , and G. Bell . 2023. “Environmental and Genetic Variation in an Asexual Plant.” Aquatic Botany 188: 103675. 10.1016/j.aquabot.2023.103675.

[ece374326-bib-0040] Jiang, K. , X. Hu , Q. Sun , et al. 2025. “Integrating Phenotypic and Molecular Approaches to Unravel Salinity and Cold Tolerance in Wetland Plants for Ecosystem Restoration.” Environmental and Experimental Botany 232: 106108. 10.1016/j.envexpbot.2025.106108.

[ece374326-bib-0041] Jost, L. 2008. “GST and Its Relatives Do Not Measure Differentiation.” Molecular Ecology 17, no. 18: 4015–4026. 10.1111/j.1365-294X.2008.03887.x. 19238703

[ece374326-bib-0042] Jueterbock, A. , C. Boström , J. A. Coyer , et al. 2020. “The Seagrass Methylome Is Associated With Variation in Photosynthetic Performance Among Clonal Shoots.” Frontiers in Plant Science 11: 571646. 10.3389/fpls.2020.571646. 33013993 PMC7498905

[ece374326-bib-0043] Kattge, J. , G. Bönisch , S. Díaz , et al. 2020. “TRY Plant Trait Database ‐ Enhanced Coverage and Open Access.” Global Change Biology 26, no. 1: 119–188. 10.1111/gcb.14904. 31891233

[ece374326-bib-0044] King, N. G. , N. J. McKeown , D. A. Smale , and P. J. Moore . 2018. “The Importance of Phenotypic Plasticity and Local Adaptation in Driving Intraspecific Variability in Thermal Niches of Marine Macrophytes.” Ecography 41: 1469–1484. 10.1111/ecog.03186.

[ece374326-bib-0045] Kosman, E. , F. Feijen , and J. Jokela . 2024. “Effective Number of Different Populations: A New Concept and How to Use It.” Ecology and Evolution 14, no. 9: e70303. 10.1002/ece3.70303. 39279796 PMC11402519

[ece374326-bib-0046] Lacoul, P. , and B. Freedman . 2006. “Environmental Influences on Aquatic Plants in Freshwater Ecosystems.” Environmental Reviews 14, no. 2: 89–136. 10.1139/A06-001.

[ece374326-bib-0047] Lambertini, C. , T. Riis , B. Olesen , J. S. Clayton , B. K. Sorrell , and H. Brix . 2010. “Genetic Diversity in Three Invasive Clonal Aquatic Species in New Zealand.” BMC Genetics 11: 52. http://www.biomedcentral.com/1471‐2156/11/52. 20565861 10.1186/1471-2156-11-52 PMC2902404

[ece374326-bib-0048] Leung, C. , D. Grulois , L. Quadrana , and L. M. Chevin . 2023. “Phenotypic Plasticity Evolves at Multiple Biological Levels in Response to Environmental Predictability in a Long‐Term Experiment With a Halotolerant Microalga.” PLoS Biology 21, no. 3: e3001895. 10.1371/journal.pbio.3001895. 36961833 PMC10075460

[ece374326-bib-0049] Li, G. , R. Li , T. Yonezawa , et al. 2024. “Conserved Genetic Background but Geographically Differentiated DNA Methylation Patterns in Invasive Alligator Weed ( *Alternanthera philoxeroides* ) Populations of China and Japan.” Biological Invasions 26, no. 7: 2351–2365. 10.1007/s10530-024-03319-0.

[ece374326-bib-0050] Lind, L. , R. L. Eckstein , and R. A. Relyea . 2022. “Direct and Indirect Effects of Climate Change on Distribution and Community Composition of Macrophytes in Lentic Systems.” Biological Reviews 97, no. 4: 1677–1690. 10.1111/brv.12858. 35388965 PMC9542362

[ece374326-bib-0051] Liu, H. , D. Yin , P. He , M. W. Cadotte , and Q. Ye . 2024. “Linking Plant Functional Traits to Biodiversity Under Environmental Change.” Biological Diversity 1, no. 1: 22–28. 10.1002/bod2.12004.

[ece374326-bib-0052] Liu, L. , J. Wang , X. Ma , et al. 2021a. “Impacts of the Yellow River and Qingtongxia Dams on Genetic Diversity of *Phragmites australis* in Ningxia Plain, China.” Aquatic Botany 169: 103341. 10.1016/j.aquabot.2020.103341.

[ece374326-bib-0053] Liu, L. , M. Yin , X. Guo , et al. 2021b. “The River Shapes the Genetic Diversity of Common Reed in the Yellow River Delta via Hydrochory Dispersal and Habitat Selection.” Science of the Total Environment 764: 144382. 10.1016/j.scitotenv.2020.144382. 33385658

[ece374326-bib-0054] Meyerson, L. A. , P. Pyšek , M. Lučanová , S. Wigginton , C. T. Tran , and J. T. Cronin . 2020. “Plant Genome Size Influences Stress Tolerance of Invasive and Native Plants via Plasticity.” Ecosphere 11, no. 5: e03145. 10.1002/ecs2.3145.

[ece374326-bib-0055] Molnár, V. , J. P. Tóth , G. Sramkó , et al. 2015. “Flood Induced Phenotypic Plasticity in Amphibious Genus Elatine (Elatinaceae).” PeerJ 3: e1473. 10.7717/peerj.1473. 26713235 PMC4690399

[ece374326-bib-0056] Morton, J. A. , C. A. Arnillas , L. Biedermann , et al. 2024. “Genome Size Influences Plant Growth and Biodiversity Responses to Nutrient Fertilization in Diverse Grassland Communities.” PLoS Biology 22, no. 12: e3002927. 10.1371/journal.pbio.3002927. 39661599 PMC11633961

[ece374326-bib-0057] Nelson, M. F. , and N. O. Anderson . 2015. “Variation Among Genotypes and Source Habitats in Growth and Fecundity of the Wetland Invasive Plant *Phalaris arundinacea* *L* .” Wetlands 35, no. 6: 1175–1184. 10.1007/s13157-015-0704-9.

[ece374326-bib-0058] Nguyen, H. M. , M. Kim , P. J. Ralph , L. Marín‐Guirao , M. Pernice , and G. Procaccini . 2020. “Stress Memory in Seagrasses: First Insight Into the Effects of Thermal Priming and the Role of Epigenetic Modifications.” Frontiers in Plant Science 11: 494. 10.3389/fpls.2020.00494. 32411166 PMC7199800

[ece374326-bib-0059] O'Brien, A. M. , J. R. Laurich , and M. E. Frederickson . 2024. “Evolutionary Consequences of Microbiomes for Hosts: Impacts on Host Fitness, Traits, and Heritability.” Evolution 78, no. 2: 237–252. 10.1093/evolut/qpad183. 37828761

[ece374326-bib-0060] Pellicer, J. , O. Hidalgo , S. Dodsworth , and I. J. Leitch . 2018. “Genome Size Diversity and Its Impact on the Evolution of Land Plants.” Genes 9, no. 2: 88. 10.3390/genes9020088. 29443885 PMC5852584

[ece374326-bib-0061] Pfennig, D. W. , and I. M. Ehrenreich . 2014. “Towards a Gene Regulatory Network Perspective on Phenotypic Plasticity, Genetic Accommodation and Genetic Assimilation.” Molecular Ecology 23, no. 18: 4438–4440. 10.1111/mec.12887. 25208504 PMC4180264

[ece374326-bib-0062] Pollux, B. J. A. , M. D. E. Jong , A. Steegh , E. Verbruggen , J. M. Van Groenendael , and N. J. Ouborg . 2007. “Reproductive Strategy, Clonal Structure and Genetic Diversity in Populations of the Aquatic Macrophyte *Sparganium emersum* in River Systems.” Molecular Ecology 16, no. 2: 313–325. 10.1111/j.1365-294X.2006.03146.x. 17217347

[ece374326-bib-0063] Rajpal, V. R. , P. Rathore , S. Mehta , et al. 2022. “Epigenetic Variation: A Major Player in Facilitating Plant Fitness Under Changing Environmental Conditions.” Frontiers in Cell and Developmental Biology 10: 1020958. 10.3389/fcell.2022.1020958. 36340045 PMC9628676

[ece374326-bib-0064] Ren, L. , X. Guo , S. Liu , et al. 2020. “Intraspecific Variation in *Phragmites australis* : Clinal Adaption of Functional Traits and Phenotypic Plasticity Vary With Latitude of Origin.” Journal of Ecology 108, no. 6: 2531–2543. 10.1111/1365-2745.13401.

[ece374326-bib-0065] Riis, T. , C. Lambertini , B. Olesen , J. S. Clayton , H. Brix , and B. K. Sorrell . 2010. “Invasion Strategies in Clonal Aquatic Plants: Are Phenotypic Differences Caused by Phenotypic Plasticity or Local Adaptation?” Annals of Botany 106, no. 5: 813–822. 10.1093/aob/mcq176. 20826438 PMC2958791

[ece374326-bib-0066] Roubeau Dumont, E. , C. Larue , H. C. Michel , et al. 2020. “Genotypes of the Aquatic Plant *Myriophyllum spicatum* With Different Growth Strategies Show Contrasting Sensitivities to Copper Contamination.” Chemosphere 245: 125552. 10.1016/j.chemosphere.2019.125552. 31846788

[ece374326-bib-0067] Sakamoto, T. , and H. Innan . 2024. “On the Evolutionary Origin of Discrete Phenotypic Plasticity.” G3: Genes, Genomes, Genetics 14, no. 9: jkae144. 10.1093/g3journal/jkae144. 38984708 PMC11373660

[ece374326-bib-0068] Sand‐Jensen, K. , K. Andersen , and T. Andersen . 1999. “Dynamic Properties of Recruitment, Expansion and Mortality of Macrophyte Patches in Streams.” International Review of Hydrobiology 84, no. 5: 497–508. 10.1002/iroh.199900044.

[ece374326-bib-0069] Schlichting, C. D. 1986. “The Evolution of Phenotypic Plasticity in Plants.” Annual Review of Ecology and Systematics 17: 667–693. https://www.jstor.org/stable/2097012.

[ece374326-bib-0070] Scorsim, B. , A. C. da Silva , L. I. Ramos , M. D. Passere , S. M. Thomaz , and A. V. de Oliveira . 2024. “Molecular Markers in Genetic Studies of Aquatic Macrophytes: A Systematic Review.” Hydrobiologia 851, no. 16: 3809–3820. 10.1007/s10750-024-05556-9.

[ece374326-bib-0071] Sheng, W. , L. Liu , Y. Wu , et al. 2024. “Exploring Salt Tolerance and Indicator Traits Across Four Temperate Lineages of the Common Wetland Plant, *Phragmites australis* .” Science of the Total Environment 912: 169100. 10.1016/j.scitotenv.2023.169100. 38086483

[ece374326-bib-0072] Smale, D. A. , and N. G. King . 2024. “Marine Macrophytes in a Changing World: Mechanisms Underpinning Responses and Resilience to Environmental Stress – An Introduction to a Virtual Issue.” New Phytologist 244: 1675–1677. 10.1111/nph.20215. 39506199

[ece374326-bib-0073] Thomaz, S. M. 2023. “Ecosystem Services Provided by Freshwater Macrophytes.” Hydrobiologia 850, no. 12–13: 2757–2777. 10.1007/s10750-021-04739-y.

[ece374326-bib-0074] Trinder, S. , O. Hidalgo , M. F. Fay , A. P. Askew , H. McAllister , and R. Whitlock . 2025. “Plant Genome Size Is Associated With Fine‐Scale Spatial Variation in Soil Depth, but Not Climatic Conditions, in the Grass *Festuca ovina* .” Ecology and Evolution 15, no. 12: e72438. 10.1002/ece3.72438. 41323070 PMC12658382

[ece374326-bib-0075] Ulukapi, K. , and A. G. Nasircilar . 2018. “Induced Mutation: Creating Genetic Diversity in Plants.” In Genetic Diversity in Plant Species ‐ Characterization and Conservation. IntechOpen. 10.5772/intechopen.81296.

[ece374326-bib-0076] Violle, C. , M.‐L. Navas , D. Vile , et al. 2007. “Let the Concept of Trait Be Functional!” Oikos 116, no. 5: 882–892. 10.1111/j.0030-1299.2007.15559.x.

[ece374326-bib-0077] Vretare, V. , S. E. B. Weisner , J. A. Strand , and W. Granéli . 2001. “Phenotypic Plasticity in *Phragmites australis* as a Functional Response to Water Depth.” Aquatic Botany 69, no. 2: 127–145. 10.1016/S0304-3770(01)00134-6.

[ece374326-bib-0078] Wei, W. , Q. Wen , H. Zhu , et al. 2025. “Effects of Phenotypic Plasticity and Genetic Variation on Plant Growth and Litter Decomposition in a Widespread Wetland Grass.” Diversity 17, no. 4: 282. 10.3390/d17040282.

[ece374326-bib-0079] Wells, C. L. , and M. Pigliucci . 2000. “Adaptive Phenotypic Plasticity: The Case of Heterophylly in Aquatic Plants.” Perspectives in Plant Ecology, Evolution and Systematics 3: 1–18. 10.1078/1433-8319-00001.

[ece374326-bib-0080] Wright, J. P. , G. M. Ames , and R. M. Mitchell . 2016. “The More Things Change, the More They Stay the Same? When Is Trait Variability Important for Stability of Ecosystem Function in a Changing Environment.” Philosophical Transactions of the Royal Society, B: Biological Sciences 371, no. 1694: 20150272. 10.1098/rstb.2015.0272. PMC4843693 27114574

[ece374326-bib-0081] Zaidem, M. L. , S. C. Groen , and M. D. Purugganan . 2019. “Evolutionary and Ecological Functional Genomics, From Lab to the Wild.” Plant Journal 97, no. 1: 40–55. 10.1111/tpj.14167. 30444573

[ece374326-bib-0082] Zhang, K. L. , Y. N. Leng , R. R. Hao , H. F. Li , M. X. Chen , and F. Y. Zhu . 2024. “Adaptation of High‐Altitude Plants to Harsh Environments: Application of Phenotypic‐Variation‐Related Methods and Multi‐Omics Techniques.” International Journal of Molecular Sciences 25, no. 23: 12666. 10.3390/ijms252312666. 39684378 PMC11641277

[ece374326-bib-0083] Zhou, W. , Z. Wang , W. Xing , and G. Liu . 2014. “Plasticity in Latitudinal Patterns of Leaf N and P of *Oryza rufipogon* in China.” Plant Biology 16, no. 5: 917–923. 10.1111/plb.12147. 24450441

